# Tocilizumab discontinuation after remission achievement in patients with adult-onset Still’s disease

**DOI:** 10.1093/rheumatology/keae179

**Published:** 2024-03-20

**Authors:** Hiroya Tamai, Yasushi Kondo, Tsutomu Takeuchi, Yuko Kaneko

**Affiliations:** Division of Rheumatology, Department of Internal Medicine, Keio University School of Medicine, Tokyo, Japan; Division of Rheumatology, Department of Internal Medicine, Keio University School of Medicine, Tokyo, Japan; Division of Rheumatology, Department of Internal Medicine, Keio University School of Medicine, Tokyo, Japan; Saitama Medical University, Iruma, Japan; Division of Rheumatology, Department of Internal Medicine, Keio University School of Medicine, Tokyo, Japan

**Keywords:** adult-onset Still’s disease, tocilizumab, drug withdrawal, remission

## Abstract

**Objectives:**

Tocilizumab, an IL-6 inhibitor, has been proven effective in patients with adult-onset Still's disease (AOSD). This study aimed to clarify whether tocilizumab can be discontinued after achieving remission and to identify factors relevant to its successful discontinuation.

**Methods:**

Consecutive patients with AOSD diagnosed according to Yamaguchi's criteria from April 2012 to July 2022, who were treated with tocilizumab, were retrospectively reviewed.

**Results:**

Forty-eight patients with AOSD treated with intravenous tocilizumab, with sufficient information, were included. Thirty-eight patients (79.2%) achieved remission after 6 months of tocilizumab treatment, 12 of whom discontinued tocilizumab during remission. Within 1 year after tocilizumab discontinuation, six patients (50.0%) recurred at a mean of 5.5 months, while the other six (50.0%) remained in remission. Between the non-recurrence and recurrence groups, no difference was found in disease activity at tocilizumab discontinuation (systemic feature score, *P* = 0.24; ferritin, *P* = 0.46). While the duration of tocilizumab use was not different (*P* = 0.32), the interval of tocilizumab administration at tocilizumab discontinuation in the recurrence group was 21 (14–35) days, which tended to be shorter than 35 (28–53) days in the non-recurrence group (*P* = 0.08). Patients with prednisolone dose <7 mg/day at last tocilizumab treatment had fewer recurrences than those without (*P* = 0.001). After recurrence, tocilizumab was resumed in half of the patients, resulting in successful disease control.

**Conclusions:**

The recurrence rate after tocilizumab discontinuation was 50% in 1 year. Patients who remained in remission with a longer interval of tocilizumab administration and lower prednisolone dose were likely to succeed in the withdrawal of tocilizumab.

Rheumatology key messages

- A total of 80% of patients with adult-onset Still's disease achieved remission with tocilizumab at 6 months.
- Half of patients recurred within one year after tocilizumab discontinuation.
- Patients in remission with longer tocilizumab interval and lower dose of prednisolone successfully withdrew tocilizumab.

## Introduction

Adult-onset Still’s disease (AOSD) is an autoinflammatory disease with systemic symptoms [1]. Although the overall prognosis is generally good, 10–20% of patients develop life-threatening complications, such as macrophage activation syndrome and disseminated intravascular coagulation syndrome [2, 3]; therefore, strict disease control is critical.

Glucocorticoids are the mainstay treatment for AOSD. Most patients with AOSD respond to glucocorticoids, but its long-term use is problematic, and many patients experience recurrence during glucocorticoid tapering. Immunosuppressive drugs that affect AOSD are required to decrease the glucocorticoid dose without causing flares [4]. Methotrexate and ciclosporin are frequently used concomitantly as immunosuppressants, although their effectiveness is empirical and limited [2, 5, 6].

Pro-inflammatory cytokines, such as IL-1, IL-6, IL-18, TNF-α and IFN-γ play important roles in the pathophysiology of AOSD [7–9]. Tocilizumab, a human anti-IL-6 receptor inhibitor, has demonstrated efficacy in suppressing systemic inflammation and decreasing glucocorticoid doses in a randomized controlled trial and in clinical practice in patients with AOSD [10–12]. Intravenous tocilizumab (8 mg/kg) is now approved for AOSD, with an administration interval arrangement permitted based on disease activity in Japan. However, the possibility of tocilizumab withdrawal has not yet been discussed.

In this study, we aimed to clarify whether tocilizumab can be discontinued after achieving remission in patients with AOSD, and to identify factors relevant to its successful discontinuation.

## Patients and methods

### Patients and data collection

We reviewed consecutive patients diagnosed with AOSD according to Yamaguchi’s criteria [13] who visited Keio University Hospital from April 2012 to July 2022. Patients who had been treated with tocilizumab were included in the study. Clinical data were collected from medical charts. The study protocol was approved by the ethics committee of Keio University School of Medicine (approval number: 20130506). The 2008 Declaration of Helsinki and the 2008 Ethical Guidelines for Clinical Research by the Japanese Ministry of Health, Labour, and Welfare were followed.

### Assessment and definitions

Disease activity was evaluated using the systemic feature score (SFS), the Pouchot score, and modified Pouchot score, as previously published [14–16]. Remission was defined as the absence of Still's disease-related symptoms, normal ESR or CRP, and no requirement for treatment intensification. Recurrence after tocilizumab discontinuation was defined as disease flare with treatment intensification for AOSD requiring either a ≥ 1.5-fold increase in glucocorticoid dose and/or initiation of a biologic agent.

### Statistical analysis

Parametric continuous values are presented as the mean and standard deviation, and non-parametric continuous values are presented as the median and interquartile range (25–75%). Continuous variables between the two groups were compared using Student’s *t* test for parametric data or the Wilcoxon signed-rank test for non-parametric data. Categorical variables were compared using Fisher’s exact test. Logistic regression analysis was used for dichotomous outcome measures, and the results were expressed as odds ratios (ORs) with 95% confidence intervals (95% CIs). Relationships between continuous variables were assessed using Pearson's correlation coefficients. An optimal cut-off value to discriminate between the two groups was determined using receiver operating characteristic (ROC) curves with sensitivity, specificity and area under the curve (AUC). The log-rank test was used to compare survival distributions between the two groups. Data from patients lost to follow-up were complemented by the last observation. All statistical analyses were performed using JMP version 16.0 (SAS Institute Inc., Cary, NC, USA). Statistical significance was set at *P* < 0.05.

## Results

### Patient flow and tocilizumab-retention rates

We identified 67 patients with AOSD who were treated with tocilizumab. After excluding 19 patients for various reasons, 48 patients were included in the analysis (Supplementary Fig. S1, available at *Rheumatology* online). Among them, 38 (79.2%) achieved remission with tocilizumab at 6 months; 13 of these patients subsequently stopped tocilizumab (12 patients while in remission and one patient because of flare), and the remaining 25 patients continued tocilizumab during the median observation period of 5.1 (IQR: 1.6–7.1) years. The timing of tocilizumab discontinuation is shown in Supplementary Fig. S2, available at *Rheumatology* online.

### Factors for remission achievement at 6 months with tocilizumab

At tocilizumab initiation, all patients except for one were treated with a mean dose of 26 ± 19 mg/day of prednisolone. Tocilizumab was administered at a median interval of 14 (IQR: 14–19) days. At 6 months, all patients except for two were still treated with prednisolone but its dose was decreased to 8.4 ± 4.6 mg/day. The median interval of tocilizumab administration was 14 (IQR: 14–28) days.

We compared the characteristics at tocilizumab initiation between patients who achieved remission at 6 months (*N* = 38) and those who did not (*N* = 10) (Supplementary Table S1, available at *Rheumatology* online). There was no significant difference in duration from initial treatment for AOSD to tocilizumab initiation (0.6 years *vs* 0.4 years, *P* = 0.61) or SFS at tocilizumab initiation (3.1 ± 1.8 *vs* 3.3 ± 1.9, *P* = 0.80), although the frequency of arthritis was higher in the non-remission group (45% *vs* 90%, *P* = 0.01). At 6 months, 60% of patients in the non-remission group still had arthritis, and 50% had skin rash. Logistic regression analysis with sex, age, arthritis and alanine aminotransferase levels at tocilizumab initiation as covariates revealed the presence of arthritis at tocilizumab initiation as a significant negative factor for remission achievement at 6 months with tocilizumab (odds ratio, 0.08 [95% CI 0.01–0.80], *P* = 0.03, Supplementary Table S2, available at *Rheumatology* online).

### Characteristics of patients who discontinued tocilizumab

To investigate the characteristics of patients who discontinued tocilizumab, we compared the characteristics at tocilizumab initiation and at 6 months between patients who discontinued tocilizumab (*N* = 13) and those who continued treatment (*N* = 25) (Supplementary Table S3, available at *Rheumatology* online). There was no significant difference in SFS between the groups at tocilizumab initiation (3.5 ± 2.2 *vs* 2.9 ± 1.6, *P* = 0.33), although the frequency of myalgia was higher in the patients who discontinued tocilizumab (39% *vs* 4%, *P* = 0.01). At 6 months, there was no significant difference between the two groups.

### Recurrence rate after tocilizumab discontinuation in remission

**Figure 1.**
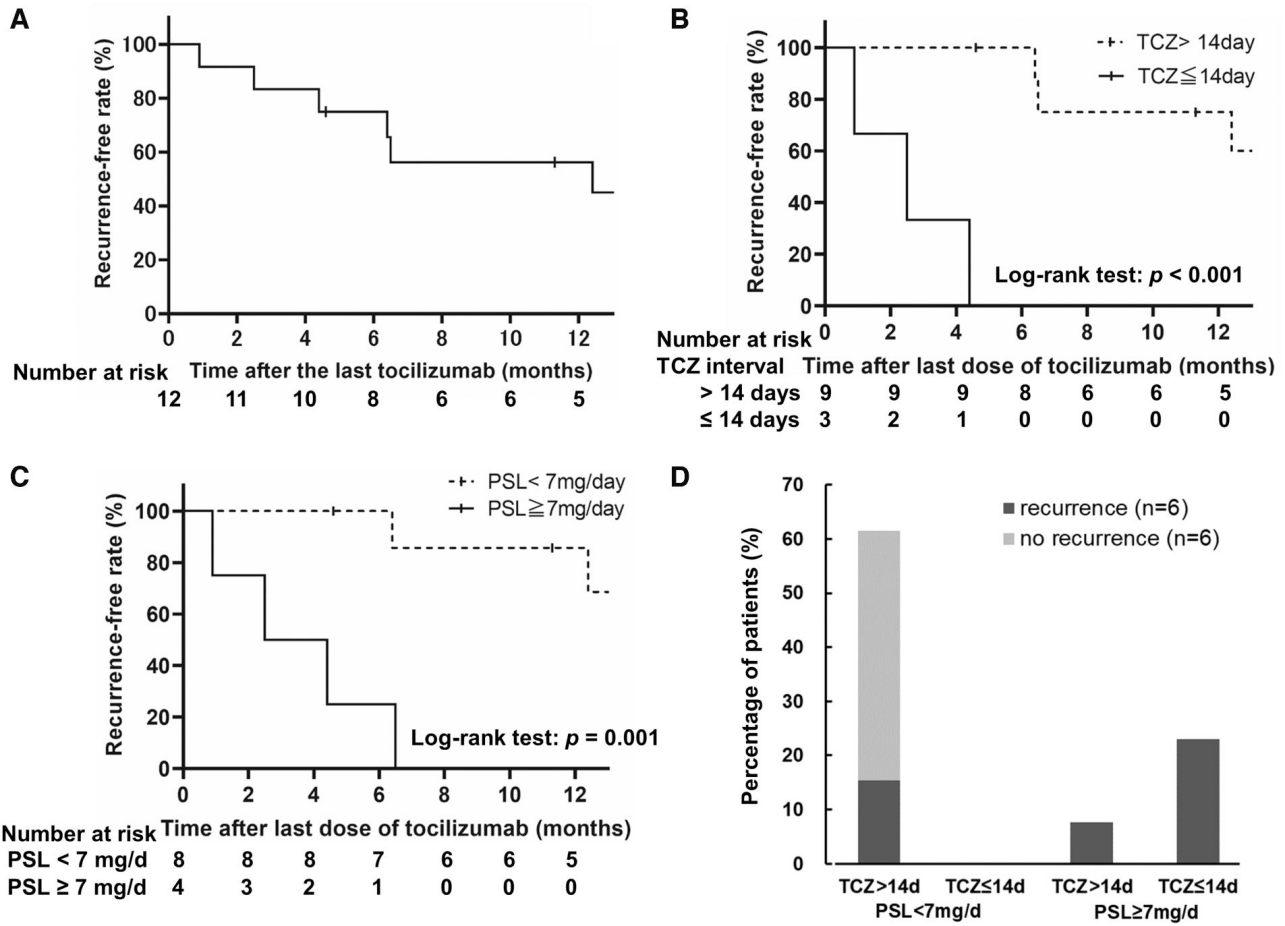
Recurrence-free rate within one year after tocilizumab discontinuation. Half of patients experienced recurrence within one year after tocilizumab discontinuation during remission (**A**). Patient with tocilizumab intervals longer than 14 days at tocilizumab discontinuation or those with prednisolone dose <7 mg/day had better recurrence-free rate than those without (**B**, **C**). Patients who did not experience recurrence within one year were treated with prednisolone <7 mg/d and with tocilizumab longer than 14 days of interval (**D**). PSL: prednisolone; TCZ: tocilizumab

We focused on the 12 patients who discontinued tocilizumab during remission. Among them, six patients (50.0%) remained in remission, while six patients (50.0%) recurred within 1 year after tocilizumab discontinuation. The mean duration from the last dose of tocilizumab to recurrence in the recurrence group was 5.5 ± 4.0 months (Fig. 1A).

The duration of tocilizumab use was not significantly different between the non-recurrence group and recurrence group (*P* = 0.32) (Table 1). A comparison of the demographic and clinical characteristics at tocilizumab initiation, 6 months with tocilizumab, and tocilizumab discontinuation between the recurrence and non-recurrence groups is shown in Table 1 and Supplementary Table S4, available at *Rheumatology* online. SFS (0.2 ± 0.4 *vs* 0.8 ± 1.2, *P* = 0.24) and serum ferritin levels (49 *vs* 17 ng/ml, *P* = 0.46) at tocilizumab discontinuation were not significantly different. On another front, the mean interval of tocilizumab administration at tocilizumab discontinuation in the recurrence group was 21 (IQR: 14–35) days, which tended to be shorter than 35 (IQR: 28–53) days in the non-recurrent group (*P* = 0.08). Glucocorticoid use was 50% in the non-recurrence group compared with 67% in the recurrence group (*P* = 1.00), and the mean dose of prednisolone was 2.7 ± 1.2 mg/day *vs* 8.5 ± 2.4 mg/day (*P* = 0.01). ROC curves depicted the cut-off of tocilizumab administration interval for successful discontinuation as 14 days with a sensitivity of 50%, specificity of 100%, and AUC of 0.81, and that of prednisolone dose as 7 mg/day with a sensitivity of 67%, specificity of 100%, and AUC of 0.75. Patients with tocilizumab interval >14 days or prednisolone dose <7 mg/day at tocilizumab discontinuation had significantly better recurrence-free rates than those without (*P* < 0.001, Fig. 1B; *P* = 0.001, Fig. 1C, respectively). No patient with tocilizumab interval ≤14 days nor who had taken ≥7 mg/day of prednisolone discontinued tocilizumab successfully, while only two of eight patients (25%) with both tocilizumab interval >14 days and prednisolone dose <7 mg/day at tocilizumab discontinuation experienced recurrence (Fig. 1D).

**Table 1.** Patients’ demographics and disease characteristics in patients with or without recurrence within 1 year after tocilizumab discontinuation

Variables	No recurrence	Recurrence	*P* value
	(*n* = 6)	(*n* = 6)	
At tocilizumab initiation			
Age, years	49.3 ± 18.4	47.5 ± 23.4	0.88
Female, *n* (%)	4 (67%)	3 (50%)	1.00
Current or past smoker, *n* (%)	3 (50%)	2 (33%)	1.00
Duration from initial treatment for AOSD, years	2.7 (0.3–19.0)	0.5 (0.1–1.2)	0.23
Recurrence before tocilizumab use	4 (67%)	3 (50.0%)	1.00
Systemic feature score	3.7 ± 1.0	3.2 ± 1.0	0.72
Clinical	1.0 ± 0.9	0.8 ± 0.8	0.73
Laboratory	2.7 ± 1.8	1.5 ± 0.6	0.73
Modified Pouchot score, *n* (%)	2.5 ± 2.0	3.2 ± 2.0	0.58
Pouchot score, *n* (%)	1.8 ± 0.6	2.2 ± 0.6	0.70
Fever, *n* (%)	3 (50%)	2 (33%)	1.00
Arthritis, *n* (%)	3 (50%)	4 (67%)	1.00
Rash, *n* (%)	2 (33%)	3 (50%)	1.00
Sore throat, *n* (%)	0	0	—
Lymphadenopathy, *n* (%)	1 (17%)	0	1.00
Hepatosplenomegaly, *n* (%)	0	0	—
Serositis, *n* (%)	0	0	—
Pneumonia, *n* (%)	0	0	—
Myalgia, *n* (%)	2 (33%)	3 (50%)	1.00
Abdominal pain, *n* (%)	0	0	—
White blood cell count, ×10^3^/μL	11283 ± 2657	12067 ± 5385	0.76
Haemoglobin, g/dL	12.3 ± 3.1	11.5 ± 2.1	0.61
Platelet count, ×10^3^/μL	270 ± 85	248 ± 108	0.71
Aspartate aminotransferase, IU/L	36 (19–148)	29 (17–110)	0.94
Alanine aminotransferase, IU/L	35 (20–274)	87 (16–156))	0.94
Abnormal liver function test, *n* (%)	3 (50%)	4 (67%)	1.00
Lactate dehydrogenase, IU/L	286 (172–477)	238 (180–521)	0.94
C-reactive protein, mg/dL	6.4 ± 8.3	4.0 ± 4.8	0.57
Erythrocyte sedimentation rate, mm/h	67.8 ± 50.5 (*n* = 5)	65.2 ± 238.6 (*n* = 5)	0.93
Ferritin, ng/mL	643 (177–2771)	345 (52–10139)	0.69
Interval of tocilizumab, days^a^	21 (14–28)	14 (14–18)	0.28
Glucocorticoid use, *n* (%)	5 (83%)	6 (100%)	1.00
Glucocorticoid dose of users, mg/day	21.1 ± 17.6	30.5 ± 20.2	0.44
Other immunosuppressants use, *n* (%)^b^	3 (50%)	2 (33%)	1.00
At tocilizumab discontinuation			
Age, years	53.0 ± 19.5	49.8 ± 22.3	0.80
Duration of tocilizumab use, years	3.8 ± 2.7	2.5 ± 1.4	0.32
Systemic feature score	0.2 ± 0.4	0.8 ± 1.2	0.24
Clinical	0 (0–0)	0 (0–0)	—
Laboratory	0.2 ± 0.4	0.8 ± 1.2	0.24
Modified Pouchot Score	0 (0–0.3)	0 (0–0)	0.40
Pouchot Score	0 (0–0.3)	0 (0–0)	0.40
Fever, *n* (%)	0	0	—
Arthritis, *n* (%)	0	0	—
Rash, *n* (%)	0	0	—
Sore throat, *n* (%)	0	0	—
Lymphadenopathy, *n* (%)	0	0	—
Hepatosplenomegaly, *n* (%)	0	0	—
Serositis, *n* (%)	0	0	—
Pneumonia, *n* (%)	0	0	—
Myalgia, *n* (%)	0	0	—
Abdominal pain, *n* (%)	0	0	—
White blood cell count, ×10^3^/μL	4933 ± 1293	8033 ± 4446	0.15
Haemoglobin, g/dL	13.4 ± 3.2	12.8 ± 2.8	0.71
Platelet count, ×10^3^/μL	183 ± 84	241 ± 91	0.28
Aspartate aminotransferase, IU/L	25.7 ± 14.1	19.3 ± 5.3	0.33
Alanine aminotransferase, IU/L	32.2 ± 32.1	15.5 ± 6.9	0.26
Abnormal liver function test, *n* (%)	1 (17%)	0	1.00
Lactate dehydrogenase, IU/L	200 ± 47	178 ± 14	0.32
C-reactive protein, mg/dL	0.03 ± 0.04	0.01 ± 0.00	0.27
Erythrocyte sedimentation rate, mm/h	6.2 ± 5.6	5.0 ± 3.8	0.68
Ferritin, ng/mL	49 (9–352)	17 (8–151)	0.46
Intravenous tocilizumab administration, *n* (%)	6 (100%)	5 (83%)	1.00
Interval of tocilizumab, days^a^	35 (28–53)	21 (14–35)	0.08
Glucocorticoid use, *n* (%)	3 (50%)	4 (67%)	1.00
Glucocorticoid dose of users, mg/day	2.7 ± 1.2	8.5 ± 2.4	0.01*
Other immunosuppressants use, *n* (%)^b^	2 (33%)	1 (17%)	1.00
Reason for discontinuation			
Planned	4 (67%)	2 (33%)	0.57
Infection	2 (33%)	2 (33%)	1.00
Malignancy	0	1 (17%)	1.00
Surgical operation	0	1 (17%)	1.00

Data are *n* (%), mean ± SD, and median (Q1–Q3).

a Converted to intravenous interval, for subcutaneous injections.

b Tacrolimus, ciclosporin, or methotrexate.

* *P *< 0.05.

AOSD: adult-onset Still’s disease.

### Factors relevant for successful tocilizumab discontinuation

In patients who experienced recurrence after tocilizumab discontinuation, there was a negative correlation trend between the prednisolone dose at tocilizumab discontinuation and the time of recurrence (r = -0.80, *P* = 0.06, Supplementary Fig. S3A, available at *Rheumatology* online) and a positive correlation between the interval of tocilizumab at tocilizumab discontinuation and the time of recurrence (r = 0.84, *P* = 0.04, Supplementary Fig. S3B, available at *Rheumatology* online). Higher prednisone doses and shorter tocilizumab intervals at tocilizumab discontinuation showed a tendency toward shorter relapse times (Supplementary Fig. S3C, available at *Rheumatology* online).

### Manifestations of AOSD recurrence

The manifestations of recurrence in patients who experienced recurrence after tocilizumab discontinuation are shown in Supplementary Table S5, available at *Rheumatology* online. The mean SFS was 4.0 ± 2.1, and CRP levels were 7.6 ± 3.2 mg/dl. Fever and arthritis were observed in 50% of patients for each. For recurrence, three (50%) patients were treated with prednisolone increased from 4.3 ± 3.8 mg/day to 33.3 ± 11.5 mg/day, and three patients (50%) were re-administrated tocilizumab. All patients achieved remission again after treatment intensification, but one patient treated with an increased dose of prednisolone alone experienced recurrence along with a decrease in the prednisolone dose.

## Discussion

Our study demonstrated that 79% of patients with AOSD could achieve remission with tocilizumab, but 50% recurred within 1 year after tocilizumab discontinuation following remission, with a mean period of 5.5 months. Patients with tocilizumab interval >14 days or a prednisolone dose <7 mg/day at tocilizumab discontinuation had significantly better recurrence-free rates. Patients with a longer interval of tocilizumab administration or a lower dose of prednisolone were likely to succeed in the withdrawal of tocilizumab.

The efficacy of IL-6 blockade has been reported in patients with refractory AOSD. In a randomized controlled trial of tocilizumab, the SFS decreased by 4.1 in the tocilizumab group compared with 2.3 in the placebo group at 12 weeks (*P* = 0.003) [10]. In an international registry, 31 refractory AOSD patients treated with tocilizumab showed significant decreases in Pouchot score from 2.0–0.0 at 6 months [17]. Our results are consistent with those of previous reports on the high disease control capability of tocilizumab.

The question of whether targeted therapy can be discontinued after remission in patients with AOSD is of great interest. In a case series of 12 months administration of intravenous tocilizumab in patients with refractory AOSD, 8 of 11 patients (73%) remained in clinical remission 6 months after the discontinuation of tocilizumab [18]. In this study, all patients who successfully stopped tocilizumab had tapered off prednisolone before tocilizumab discontinuation, whereas the other patients who relapsed, had continued prednisolone at the last tocilizumab dose. A case report described two patients who discontinued tocilizumab after an extended interval of up to 8 weeks or after tapering the dose of tocilizumab to 2 mg/kg, although the latter relapsed 11 months later while using 10 mg/day of prednisolone [19]. Our study showed that patients with tocilizumab interval >14 days or prednisolone dose <7 mg/day at tocilizumab discontinuation had significantly fewer recurrences than those without. Taken together, stable conditions with extended intervals of tocilizumab administration and very low doses of concomitant glucocorticoids are essential for successful discontinuation.

Our study has some limitations. First, the results were obtained from a retrospective cohort study with a small sample size. The decision to initiate or discontinue tocilizumab was at the discretion of the attending physicians, which may have caused a selection bias. Second, a consensus definition of remission or recurrence for AOSD has not yet been established, which might make comparison with other studies difficult. Despite these limitations, our study provides important insights for clinical practice and warrants further research.

In conclusion, our study revealed high remission achievement rates of tocilizumab in patients with AOSD, as well as a high recurrence rate after tocilizumab discontinuation. Persistent remission with a longer interval of tocilizumab administration and a lower dose of prednisolone are key factors in the successful withdrawal of tocilizumab. Further studies are required to confirm these findings.

## Supplementary material

Supplementary material is available at *Rheumatology* online.

## Supplementary Material

keae179_Supplementary_Data

## Data Availability

The data that underlying this article cannot be shared publicly due to the privacy of individuals who participated in the study. The data will be shared if a reasonable request is made to the corresponding author.
