# Supplementary material for: Tocilizumab discontinuation after remission achievement in patients with adult-onset Still’s disease
Source: Rheumatology (Oxford). 2024 Mar 20;64(3):1437–42. doi: 10.1093/rheumatology/keae179 (PMC11879347; doi:10.1093/rheumatology/keae179)
Supplement: keae179_Supplementary_Data [file keae179_supplementary_data.pdf]

Supplementary Material

Supplementary Figure S1. Patients' flow.

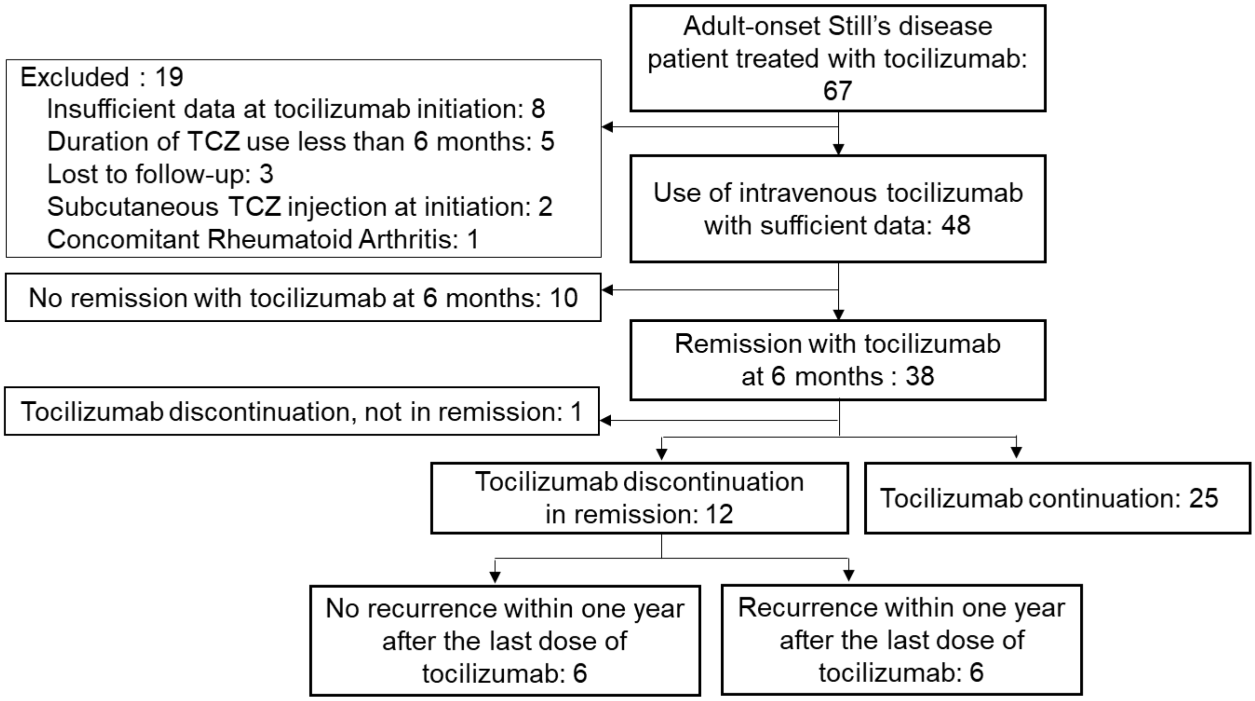

A total of 67 patients had a history of tocilizumab use and 48 patients were included in the study. Among 38 patients who achieved remission at six months of treatment with tocilizumab, 12 patients discontinued tocilizumab in remission and six patients recurred within one year.

Supplementary Figure S2. Timing of tocilizumab discontinuation.

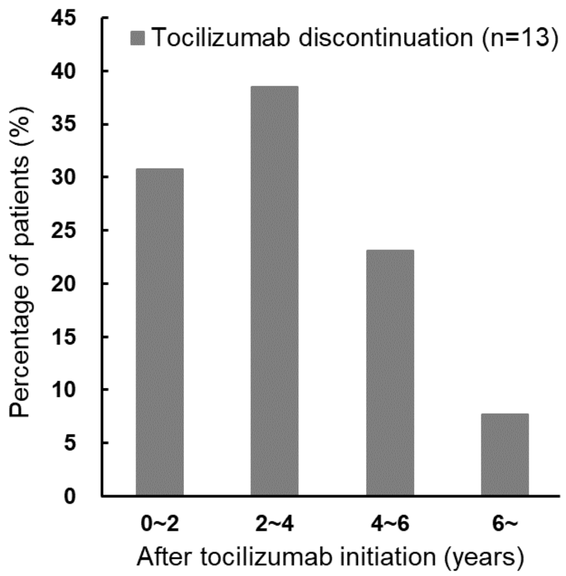

In 13 patients who discontinued tocilizumab, four had discontinued tocilizumab within 2 years of initiation, five between 2 and 4 years, three between 4 and 6 years, and one after more than 6 years.

Supplementary Figure S3. Correlation between the interval of tocilizumab or dose of prednisolone at tocilizumab discontinuation and the time of recurrence after tocilizumab discontinuation.

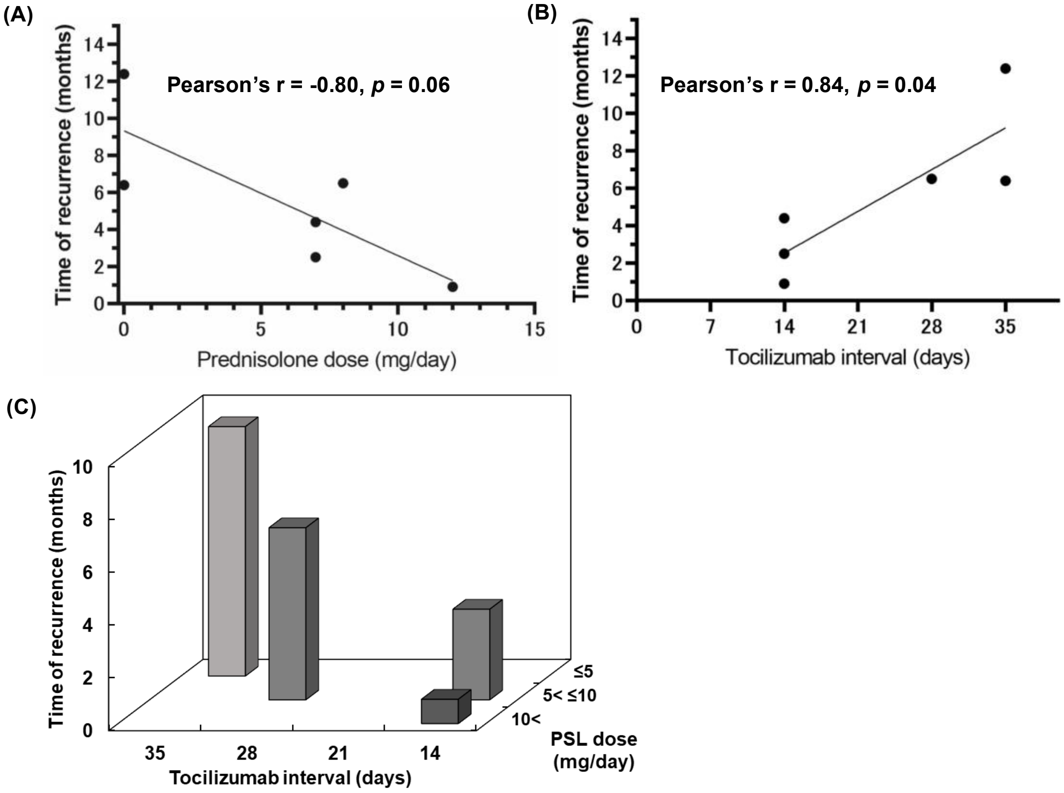

There was a negative correlation trend between the prednisolone dose at the tocilizumab and the time of recurrence (A), and positive correlation between the interval of tocilizumab at the last dose of tocilizumab and the time of recurrence (B). Higher prednisone doses and shorter tocilizumab intervals at tocilizumab discontinuation showed a tendency toward shorter time to relapse (C).

Supplementary Table S1. Patients' demographics and disease characteristics of patients with or without remission at six months of tocilizumab treatment.

Variables	All (n = 48)	Remission (n = 38)	No remission (n = 10)	P value
**At tocilizumab initiation**				
Age, years	47±17	45±17	51±18	0.36
Female, n (%)	35 (73%)	26 (68%)	9 (90%)	0.25
Current or past smoker, n (%)	21 (45%)	16 (42%)	5 (56%)	0.49
Duration from initial treatment for AOSD, years	0.5 (0.1-2.0)	0.6 (0.1-2.3)	0.4 (0.1-1.6)	0.61
Recurrence before tocilizumab use, n (%)	25 (52%)	19 (50%)	6 (60%)	0.73
Systemic feature score	3.2±1.8	3.1±1.8	3.3±1.9	0.80
Clinical	1.0±0.9	1.0±1.0	1.1±0.7	0.82
Laboratory	2.1±1.4	2.1±1.4	2.2±1.8	0.86
Modified Pouchot Score, n (%)	2.5±1.8	2.5±1.9	2.6±1.3	0.84
Pouchot Score, n (%)	1.9±1.5	1.9±1.5	1.7±1.3	0.71
Fever, n (%)	21 (44%)	17 (45%)	4 (40%)	1.00
Arthritis, n (%)	26 (54%)	17 (45%)	9 (90%)	0.01*
Rash, n (%)	21 (44%)	16 (42%)	5 (50%)	0.73
Sore throat, n (%)	4 (8%)	3 (8%)	1 (10%)	1.00
Lymphadenopathy, n (%)	5 (10%)	3 (8%)	2 (20%)	0.28
Hepatosplenomegaly, n (%)	3 (6%)	3 (8%)	0	1.00
Serositis, n (%)	0	0	0	–
Pneumonia, n (%)	0	0	0	–
Myalgia, n (%)	6 (13%)	6 (16%)	0	0.32
Abdominal pain, n (%)	0	0	0	–
White blood cell count, $\times 10^3/\mu L$	12549±6397	12748±6000	11790±8053	0.68
Hemoglobin, g/dL	12.2±2.1	12.3±2.2	11.6±1.4	0.37
Platelet count, $\times 10^3/\mu L$	284±108	282±108	292±115	0.80

Aspartate aminotransferase, IU/L	23 (16-39)	22 (17-41)	23 (16-34)	0.71
Alanine aminotransferase, IU/L	25 (15-63)	27 (16-70)	16 (14-23)	0.04*
Abnormal liver function test, n (%)	18 (38%)	16 (42%)	2 (20%)	0.28
Lactate dehydrogenase, IU/L	274 (179-384)	245 (177-389)	301 (238-354)	0.61
C-reactive protein, mg/dL	3.9±5.1	4.0±5.3	3.3±4.8	0.67
Ferritin, ng/mL	535 (60-1605)	535 (58-1686)	798 (63-1519)	1.00
Interval of tocilizumab, days †	14 (14-19)	14 (14-28)	14 (14-16)	0.67
Glucocorticoid use, n (%)	47 (98%)	37 (97%)	10 (100%)	1.00
Glucocorticoid dose of users, mg/day	26±19	28±20	19±15	0.20
Other immunosuppressants use, n (%) ‡	16 (33%)	14 (37%)	2 (20%)	0.46
**At 6 months of tocilizumab treatment**				
Duration of tocilizumab use, months	6.0±0.4	6.0±0.4	5.9±0.3	0.66
Systemic feature score	0(0-1)	0 (0-0.3)	1 (0-1.3)	0.005*
Clinical	0 (0-0)	0 (0-0)	1 (0-1)	<0.001*
Laboratory	0 (0-0)	0 (0-0.3)	0 (0-0.3)	0.79
Modified Pouchot Score	0 (0-1)	0 (0-0)	1.5 (1.0-2.3)	<0.001*
Pouchot Score	0 (0-1)	0 (0-0)	1.0 (0-2.0)	0.001*
Fever, n (%)	3 (6%)	0	3 (30%)	0.01*
Arthritis, n (%)	6 (13%)	0	6 (60%)	<0.001*
Rash, n (%)	5 (10%)	0	5 (50%)	0.0001*
Sore throat, n (%)	1 (2%)	0	1 (10%)	0.21
Lymphadenopathy, n (%)	0	0	0	–
Hepatosplenomegaly, n (%)	0	0	0	–
Serositis, n (%)	0	0	0	–
Pneumonia, n (%)	0	0	0	–
Myalgia, n (%)	0	0	0	–

Abdominal pain, n (%)	0	0	0	–
White blood cell count, ×10^3/μL	7600±4753	7666±4906	7350±4353	0.85
Hemoglobin, g/dL	13.3±1.9	13.5±2.0	12.8±1.0	0.17
Platelet count, ×10^3/μL	214±57	216±56	204±64	0.54
Aspartate aminotransferase, IU/L	19 (16-29)	19 (16-27)	21 (16-30)	1.00
Alanine aminotransferase, IU/L	18 (12-28)	19 (13-29)	15 (11-28)	0.53
Abnormal liver function test, n (%)	6 (13%)	5 (13%)	1 (10%)	1.00
Lactate dehydrogenase, IU/L	209 (183-283)	305 (184-284)	216 (170-306)	0.84
C-reactive protein, mg/dL	0.01 (0.01-0.02)	0.01 (0.01-0.01)	0.02 (0.01-0.03)	0.06
Ferritin, ng/mL	44 (14-101)	42 (14-96)	76 (19-108)	0.55
Intravenous tocilizumab administration, n (%)	46 (96%)	36 (95%)	10 (100%)	1.00
Interval of tocilizumab, days †	14 (14-28)	18 (14-28)	14 (14-16)	0.09
Glucocorticoid use, n (%)	46 (96%)	36 (95%)	10 (100%)	1.00
Glucocorticoid dose of users, mg/day	8.4±4.6	8.4±4.5	8.2±5.4	0.86
Other immunosuppressants use, n (%) ‡	15 (31%)	12 (32%)	3 (30%)	1.00

Data are n (%), mean ± SD, and median (Q1-Q3). AOSD=Adult-onset Still's Disease.
† Converted to intravenous interval, for subcutaneous injections
‡ Tacrolimus, cyclosporine or methotrexate
p value was calculated between remission and no remission group
* $p < 0.05$

Supplementary Table S2. Logistic regression analyses for remission achievement at six months with tocilizumab treatment.

	Univariable		Multivariable	
**Variables**	OR (95% CI)	*p*	OR (95% CI)	*p*
Female	0.24 (0.03-2.12)	0.200	0.23 (0.02-2.54)	0.228
Age at tocilizumab initiation, year	0.98 (0.94-1.02)	0.352	0.99 (0.95-1.04)	0.697
SFS at tocilizumab initiation	0.96 (0.65-1.39)	0.795		
Arthritis at tocilizumab initiation, n (%)	0.09 (0.01-0.78)	0.029*	0.08 (0.01-0.80)	0.032*
AST at tocilizumab initiation, IU/L	1.02 (0.98-1.06)	0.343		
ALT at tocilizumab initiation, IU/L	1.04 (0.99-1.10)	0.130	1.06 (0.99-1.12)	0.101
Tocilizumab interval at initiation, days †	1.06 (0.93-1.22)	0.380		

ALT=Alanine aminotransferase; AST=Aspartate aminotransferase; OR=Odds ratio; SFS= Systemic feature score.
† Converted to intravenous interval, for subcutaneous injections
* $p < 0.05$

Supplementary Table S3. Patients' demographics and disease characteristics of patients with or without tocilizumab discontinuation.

Variables	Tocilizumab discontinuation (n=13)	Tocilizumab continuation (n = 25)	P value
**At tocilizumab initiation**			
Age, years	48.3±19.2	44.0±15.5	0.46
Female, n (%)	8 (62%)	18 (72%)	0.71
Current or past smoker, n (%)	6 (46%)	10 (40%)	0.74
Duration from initial treatment for AOSD, years	1.1 (0.3-10.2)	0.5 (0.1-1.4)	0.15
Recurrence before tocilizumab use, n (%)	8 (62%)	11 (44%)	0.50
Modified Pouchot Score, n (%)	2.9±1.9	2.2±1.8	0.29
Pouchot Score, n (%)	2.1±1.4	1.8±1.6	0.60
Systemic feature score	3.5±2.2	2.9±1.6	0.33
Clinical	0.9±0.8	1.1±1.1	0.64
Laboratory	2.6±1.6	1.8±1.2	0.10
Fever, n (%)	6 (46%)	11 (44%)	1.00
Arthritis, n (%)	8 (62%)	9 (36%)	0.18
Rash, n (%)	5 (38%)	11 (44%)	1.00
Sore throat, n (%)	0	3 (12%)	0.54
Lymphadenopathy, n (%)	1 (8%)	2 (8%)	1.00
Hepatosplenomegaly, n (%)	0	3 (12%)	0.54
Serositis, n (%)	0	0	–
Pneumonia, n (%)	0	0	–
Myalgia, n (%)	5 (39%)	1 (4%)	0.01*
Abdominal pain, n (%)	0	0	–
White blood cell count, ×10^3/μL	13538±7767	12337±4983	0.56
Hemoglobin, g/dL	12.0±2.4	12.5±2.1	0.51
Platelet count, ×10^3/μL	273±102	287±112	0.70

Aspartate aminotransferase, IU/L	32 (19.5-89.5)	19 (15.5-32)	0.06
Alanine aminotransferase, IU/L	69 (20-144)	25 (15-44)	0.08
Abnormal liver function test, n (%)	8 (62%)	8 (32%)	0.10
Lactate dehydrogenase, IU/L	353±291	284±130	0.43
C-reactive protein, mg/dL	5.29±6.31	3.40±4.66	0.30
Ferritin, ng/mL	549 (101-3386)	495 (33-1566) (n=24)	0.37
Interval of tocilizumab, days †	14 (14-28)	14 (14-21)	0.46
Glucocorticoid use, n (%)	12 (92%)	25 (100%)	0.34
Glucocorticoid dose of users, mg/day	26.5±5.8	28.5±4.0	0.79
Other immunosuppressants use, n (%) ‡	5 (38%)	9(36%)	1.00
**At 6 months of tocilizumab treatment**			
Duration of tocilizumab use, months	5.9±0.5	6.0±0.3	0.36
Modified Pouchot Score	0 (0-0)	0 (0-1)	0.56
Pouchot Score	0 (0-0)	0 (0-1)	0.56
Systemic feature score	0 (0-1)	0 (0-0)	0.42
Clinical	0	0	–
Laboratory	0 (0-1)	0 (0-0)	0.42
Fever, n (%)	0	0	–
Arthritis, n (%)	0	0	–
Rash, n (%)	0	0	–
Sore throat, n (%)	0	0	–
Lymphadenopathy, n (%)	0	0	–
Hepatosplenomegaly, n (%)	0	0	–
Serositis, n (%)	0	0	–
Pneumonia, n (%)	0	0	–
Myalgia, n (%)	0	0	–
Abdominal pain, n (%)	0	0	–
White blood cell count, $\times 10^3/\mu L$	7123±5577	7948±4616	0.63
Hemoglobin, g/dL	13.2±2.4	13.6±1.9	0.62

Platelet count, $\times 10^3/\mu L$	21.8±7.2	21.5±4.8	0.87
Aspartate aminotransferase, IU/L	22.4±4.1	25.7±2.9	0.52
Alanine aminotransferase, IU/L	21.5±5.3	25.4±3.8	0.55
Abnormal liver function test, n (%)	1 (8%)	4 (16%)	0.64
Lactate dehydrogenase, IU/L	221±55	238±67	0.44
C-reactive protein, mg/dL	0.06±0.15	0.02±0.04	0.84
Ferritin, ng/mL	53 (13-196)	40 (15-93)	0.42
Intravenous tocilizumab administration, n (%)	12 (92%)	24 (96%)	1.00
Interval of tocilizumab, days †	14 (14-32)	21 (14-28)	0.62
Glucocorticoid use, n (%)	12 (92%)	24 (96%)	1.00
Glucocorticoid dose of users, mg/day	8.2±1.3	8.6±0.9	0.80
Other immunosuppressants use, n (%) ‡	3 (23%)	9 (36%)	0.49

Data are n (%), mean ± SD, and median (Q1-Q3). AOSD=Adult-onset Still's Disease.
† Converted to intravenous interval, for subcutaneous injections
‡ Tacrolimus, cyclosporine or methotrexate
* $p < 0.05$

Supplementary Table S4. Patients' demographics and disease characteristics at six months of tocilizumab treatment in patients with or without recurrence within 1 year after tocilizumab discontinuation.

Variables	No recurrence (n = 6)	Recurrence (n = 6)	P value
**At 6 months of tocilizumab treatment**			
Duration of tocilizumab use, months	6.1±0.3	5.7±0.7	0.19
Remission, n (%)	6 (100%)	6 (100%)	–
Systemic feature score	0 (0-0.3)	0 (0-1.5)	0.53
Clinical	0 (0-0)	0 (0-0)	–
Laboratory	0 (0-0.3)	0 (0-1.5)	0.53
Modified Pouchot Score	0 (0-0.3)	0 (0-0)	0.40
Pouchot Score	0 (0-0.3)	0 (0-0)	0.40
Fever, n (%)	0	0	–
Arthritis, n (%)	0	0	–
Rash, n (%)	0	0	–
Sore throat, n (%)	0	0	–
Lymphadenopathy, n (%)	0	0	–
Hepatosplenomegaly, n (%)	0	0	–
Serositis, n (%)	0	0	–
Pneumonia, n (%)	0	0	–
Myalgia, n (%)	0	0	–
Abdominal pain, n (%)	0	0	–
White blood cell count, $\times 10^3/\mu L$	4983±1789	6700±4295	0.39
Hemoglobin, g/dL	14.1±2.5	12.2±2.2	0.19
Platelet count, $\times 10^3/\mu L$	182±53	249±82	0.13
Aspartate aminotransferase, IU/L	25.7±15.6	19.8±5.7	0.42
Alanine aminotransferase, IU/L	29.8±26.0	13.5±4.5	0.19
Abnormal liver function test, n (%)	1 (17%)	0	1.00
Lactate dehydrogenase, IU/L	225±50	199±41	0.35

C-reactive protein, mg/dL	0.01 (0.01-0.01)	0.01 (0.01-0.15)	0.40
Erythrocyte sedimentation rate, mm/hr	3.8±1.9	5.6±3.8 (n = 5)	0.34
Ferritin, ng/mL	49 (14-318)	52 (10-134)	0.58
Intravenous tocilizumab administration, n (%)	6 (100%)	5 (83%)	1.00
Interval of tocilizumab, days †	28 (25-37)	14 (14-19)	0.07
Glucocorticoid use, n (%)	5 (83%)	6 (100%)	1.00
Glucocorticoid dose of users, mg/day	6.2±3.3	7.8±4.4	0.51
Other immunosuppressants use, n (%) ‡	2 (33%)	1 (17%)	1.00

Data are n (%), mean ± SD, and median (Q1-Q3).
† Converted to intravenous interval, for subcutaneous injections
‡ Tacrolimus, cyclosporine or methotrexate
* $p < 0.05$

Supplementary Table S5. Manifestations at recurrence within one year after tocilizumab discontinuation.

Variables	Recurrence (n=6)
**At recurrence**	
Time from the last use of tocilizumab, months	5.5±4.0
Systemic feature score	4.0±2.1
Clinical	1.0±1.3
Laboratory	3.0±1.5
Modified Pouchot Score	2.5±2.0
Pouchot Score	2.0±1.8
Fever, n (%)	3 (50%)
Arthritis, n (%)	3 (50%)
Rash, n (%)	1 (17%)
Sore throat, n (%)	1 (17%)
Lymphadenopathy, n (%)	1 (17%)
Hepatosplenomegaly, n (%)	1 (17%)
Serositis, n (%)	0
Pneumonia, n (%)	0
Myalgia, n (%)	2 (33%)
Abdominal pain, n (%)	0
White blood cell count, $\times 10^3/\mu L$	13417±7434
Hemoglobin, g/dL	11.0±2.6
Platelet count, $\times 10^3/\mu L$	325±104
Aspartate aminotransferase, IU/L	47.7±63.1
Alanine aminotransferase, IU/L	64.3±99.0
Abnormal liver function test, n (%)	2 (33%)
Lactate dehydrogenase, IU/L	208±56
C-reactive protein, mg/dL	7.6±3.2
Ferritin, ng/mL	185 (36-342)

Glucocorticoid use, n (%)	4 (67%)
Glucocorticoid dose of users, mg/day	7.0±2.2
Other immunosuppressants use‡	1 (17%)
**After recurrence**	
Glucocorticoid dose increase	3 (50%)
Glucocorticoid dose of increased user, mg/day	33.3±11.5
Re-administration of tocilizumab	3 (50%)
Adding Other biologics	1 (17%)

Data are n (%), mean ± SD, and median (Q1-Q3).
‡ Tacrolimus, cyclosporine or methotrexate
